# Assessment of fitness and vector competence of a New Caledonia *w*Mel *Aedes aegypti* strain before field-release

**DOI:** 10.1371/journal.pntd.0009752

**Published:** 2021-09-07

**Authors:** Nicolas Pocquet, Olivia O’Connor, Heather A. Flores, Jordan Tutagata, Morgane Pol, David J. Hooker, Catherine Inizan, Sylvie Russet, Johanna M. Duyvestyn, Etiene C. Pacidônio, Dominique Girault, Daniela da Silva Gonçalves, Marine Minier, Frédéric Touzain, Elodie Chalus, Kevin Lucien, Florie Cheilan, Tristan Derycke, Sylvie Laumond, Cameron P. Simmons, Myrielle Dupont-Rouzeyrol, Nadège Rossi

**Affiliations:** 1 URE Medical Entomology, Institut Pasteur of New Caledonia, Noumea, New Caledonia; 2 URE Dengue and Arboviruses, Institut Pasteur of New Caledonia, Noumea, New Caledonia; 3 World Mosquito Program, Institute of Vector-Borne Disease, Monash University, Melbourne, Victoria, Australia; 4 World Mosquito Program, Institut Pasteur of New Caledonia, Noumea, New Caledonia; 5 Service de Transfusion Sanguine/Centre de Don du Sang, Centre Hospitalier Territorial, Noumea, New Caledonia; 6 Mairie de Nouméa, Noumea, New Caledonia; 7 Direction des Affaires Sanitaires et Sociales, Noumea, New Caledonia; 8 Oxford University Clinical Research Unit, Ho Chi Minh City, Vietnam; 9 Nuffield Department of Medicine, University of Oxford, Oxford, United Kingdom; INDEPENDENT RESEARCHER, UNITED STATES

## Abstract

**Background:**

Biological control programs involving *Wolbachia*-infected *Aedes aegypti* are currently deployed in different epidemiological settings. New Caledonia (NC) is an ideal location for the implementation and evaluation of such a strategy as the only proven vector for dengue virus (DENV) is *Ae*. *aegypti* and dengue outbreaks frequency and severity are increasing. We report the generation of a NC *Wolbachia-*infected *Ae*. *aegypti* strain and the results of experiments to assess the vector competence and fitness of this strain for future implementation as a disease control strategy in Noumea, NC.

**Methods/principal findings:**

The NC *Wolbachia* strain (NC-*w*Mel) was obtained by backcrossing Australian AUS-*w*Mel females with New Caledonian Wild-Type (NC-WT) males. Blocking of DENV, chikungunya (CHIKV), and Zika (ZIKV) viruses were evaluated via mosquito oral feeding experiments and intrathoracic DENV challenge. Significant reduction in infection rates were observed for NC-*w*Mel *Ae*. *aegypti* compared to WT *Ae*. *aegypti*. No transmission was observed for NC-*w*Mel *Ae*. *aegypti*. Maternal transmission, cytoplasmic incompatibility, fertility, fecundity, wing length, and insecticide resistance were also assessed in laboratory experiments. *Ae*. *aegypti* NC-*w*Mel showed complete cytoplasmic incompatibility and a strong maternal transmission. *Ae*. *aegypti* NC-*w*Mel fitness seemed to be reduced compared to NC-WT *Ae*. *aegypti* and AUS-*w*Mel *Ae*. *aegypti* regarding fertility and fecundity. However further experiments are required to assess it accurately.

**Conclusions/significance:**

Our results demonstrated that the NC-*w*Mel *Ae*. *aegypti* strain is a strong inhibitor of DENV, CHIKV, and ZIKV infection and prevents transmission of infectious viral particles in mosquito saliva. Furthermore, our NC-*w*Mel *Ae*. *aegypti* strain induces reproductive cytoplasmic incompatibility with minimal apparent fitness costs and high maternal transmission, supporting field-releases in Noumea, NC.

## Author summary

Dengue represents a risk for almost half of the world’s population, especially throughout the tropics. In New Caledonia, dengue outbreaks have become more frequent in the past decade along with the recent circulation of chikungunya and Zika viruses. The opportunity to use the biocontrol method involving the release of *Wolbachia*-infected *Ae*. *aegypti* mosquitoes has been investigated as an alternative solution to the traditional control methods, like elimination of larval habitats and pyrethroid insecticide application to kill adults, which are becoming insufficient. A local strain of *Ae*. *aegypti* carrying *Wolbachia* (NC-*w*Mel) has been generated and tested to evaluate its pathogen blocking capacity for the four dengue virus serotypes as well as chikungunya and Zika viruses. The fitness of NC-*w*Mel strain has also been assessed to estimate its ability to compete with the wild-type strain in the field. Noumea city, where a third of the population of New Caledonia resides, has been chosen as the first site to implement the method in New Caledonia. As *Ae*. *aegypti* is the only proven vector in New Caledonia, we expect a significant impact on dengue outbreaks occurring in Noumea as soon as a high frequency of NC-*w*Mel is established in the population.

## Introduction

With an estimated 390 million infected people per year, dengue still represents a major public health problem throughout the tropics [1]. Dengue viruses (DENVs) are transmitted to humans by the bite of infected mosquitoes from the genus *Aedes*, with *Aedes aegypti* being the predominate vector. Dengue infection is a re-emerging disease caused by dengue virus (DENV) belonging to the genus *Flavivirus*. DENVs are divided in four serotypes (DENV-1 to -4), themselves subdivided in genotypes. Infection with one serotype is thought to provide life-long protection from reinfection with the same serotype but does not prevent secondary infection by another serotype [1,2]. The spectrum of dengue clinical presentations is broad, ranging from asymptomatic to severe, sometimes fatal infections [2].

New Caledonia (NC), a French island territory located in the subtropical Pacific region with a population of approximately 280,000, has a history of recurrent dengue outbreaks. In the past decade, DENV circulation has increased in NC, causing recurrent outbreaks with cases detected every year [3] along with chikungunya virus (CHIKV) and Zika virus (ZIKV) circulation [3]. During the three last major DENV outbreaks in 2008–2009, 2012–2013, and 2016–2018, the NC Health Authorities reported 9,589, 11,240, and 7,266 DENV cases, respectively [3,4]. To date in NC, the only proven vector for DENV is *Aedes aegypti*. Until recently, the only means of controlling dengue were based on regular public prevention campaigns and vector control measures, consisting of elimination of larval habitats and pyrethroid insecticide application to kill adults. Although these campaigns have decreased the number of larval habitats and mosquitoes [3], this decrease has not been sufficient to prevent dengue circulation [3]. Furthermore, the low efficiency of outdoor space spraying and resistance to the pyrethroid deltamethrin has reduced the efficacy of control of *Ae*. *aegypti* adults in Noumea [5].

Given these concerns, population introgression strategy, based on the release of *Wolbachia*-infected *Ae*. *aegypti* mosquitoes in the environment, has been identified as a promising strategy to control dengue in NC. *Wolbachia* is a Gram-negative bacterium mostly present in arthropods with more than 40–65% of insect species harbouring *Wolbachia* [6,7]. Mainly transmitted vertically, this bacterium can manipulate the host reproduction in order to maximize its maternal transmission (MT) through the eggs. Females are favored by *Wolbachia* through parthenogenesis, feminization, male-killing, and cytoplasmic incompatibility (CI) [8]. CI is the most common alteration and occurs when *Wolbachia*-infected males mate with uninfected females, leading to death of embryos from uninfected eggs, which promotes the spread of *Wolbachia* and its maintenance in mosquito populations [9]. *Wolbachia*-infected females can rescue the lethality, providing them with a reproductive advantage over uninfected females [10]. *Wolbachia* can also alter responses to infections to reduce arbovirus transmission. *Wolbachia* transinfection into *Ae*. *aegypti* thus limits infection with DENV, CHIKV, and ZIKV [11–15]. Combining their ability to invade the host population by inducing CI and to interfere negatively with the transmission of viruses, *Wolbachia* has been deployed to prevent the transmission of mosquito-borne diseases. The goal is to establish *Wolbachia* in wild mosquito vector populations and to interrupt local virus transmission from mosquitoes to humans as *Ae*. *aegypti* carrying *Wolbachia* have a lower transmission potential for arboviruses [11,15].

This method is potentially applicable to NC for the following reasons: (i) the targeted mosquito species is *Ae*. *aegypti*, which is the only known DENV vector in NC to date [16], (ii) the method has already shown its efficiency elsewhere [17,18], (iii) the implementation of this method is considered to be safe for humans, animals, and the environment [19]. Finally, (iv) this method is self-sustaining through the CI and MT phenotypes which promote the maintenance of *Wolbachia* in mosquito populations [20].

As highlighted by [10], before being released, the *Wolbachia*-transinfected *Ae*. *aegypti* strain has to be tested to ensure its strong protection against virus replication, to demonstrate high levels of *Wolbachia* MT as well as CI. Even if the majority of the studies have shown no or low fitness impacts of *w*Mel in *Aedes aegypti* [21], main fitness parameters have to be assessed to ensure that *Ae*. *aegypti* carrying *Wolbachia* will not be disadvantaged during the introgression in the environment. Here we report the generation of a *Wolbachi*a-transinfected Noumea strain of *Ae*. *aegypti* and present results of laboratory experiments to assess its vector competence for DENVs, CHIKV and ZIKV. *Wolbachia* MT and CI, as well as fitness determinants (fertility, fecundity, and wing length) and insecticide resistance status were also evaluated.

## Methods

### Ethics statement

In NC, human blood for mosquito rearing and artificial blood feeding experiments were obtained from blood donor center (Service de Transfusion Sanguine, NC Hospital), upon consent of patients. Ethical approval was granted by the Consultative Ethics Committee of New Caledonia 16.03.2017. Ethical approval for the collection of mosquitoes from Noumea was granted by authorities from the South Province of New Caledonia (ordinance No. 1415-2019/ARR/DENV). Ethical approval for reusing serum samples received administrative and ethical clearance in France from the “Comité de Protection des Personnes Sud-Est II” (n° ID-RCB 2019-A03114-53, n° CPP 19.12.06.49357) and by the Consultative Ethics Committee of New Caledonia. At Monash University, mosquito colonies were blood fed on the arms of adult, human volunteers in accordance with Monash University Human Research Ethics permit number CF11/0766-2011000387. Written informed consent was provided by all volunteers prior to commencement.

### Mosquito rearing

All strains were reared and maintained in a controlled laboratory environment, at 28°C ± 1°C and 80% ± 10% relative humidity, with a 12:12 light: dark photoperiod in NC, and at 26°C ± 1°C with 65% ± 10% relative humidity and a 12 h:12 h light: dark photoperiod in Australia (mosquitoes used for intrathoracic injection experiments only). A 10% sucrose solution was provided to adults. Females were blood-fed with human blood twice a week (every 3 or 4 days) with artificial membrane feeding systems (Hemotek, United Kingdom). Eggs oviposited on cups lined with filter-paper were removed at each blood feeding and kept in a humid atmosphere for 48 h to allow embryos to fully develop before being dried. Hatching solution comprising one litter of pre-boiled water and 0.2 g of tetramin (Tetra, Melle, Germany) was prepared 24 h ahead of time. Synchronous hatching was induced by placing eggs in the hatching solution for 24 h, at room temperature. Then, larvae were allocated in trays to obtain a density of 100 larvae for 1.5 L of tap water. Larvae were fed *ad libitum* with a mix of 10% of yeast and 90% of Protinova (defatted dry powder made of *Hermetia illucens* larvae, supplied by Innova Feed).

### Mosquito strains

The origin, status of *Wolbachia* infection and use of each strain are summarized in Table 1. The NC wild type strain (NC-WT) was established from larvae collected twice in the field at the same location, in Noumea, NC, in June and November 2018 (978 and 1526 larvae collected, respectively). Each time, this strain was maintained under laboratory conditions for a maximum of two generations to maintain genetic diversity and limit the impacts of inbreeding.

**Table 1:** Origin, status of *Wolbachia* infection and use of *Aedes aegypti* strains.

Strain	Origin	Year of collection/creation	*Wolbachia* infection status	Type of experiment	Reference
NC-WT	Noumea, New Caledonia	2018	-	BC, AIBF, IT, FC, IR	This study
NC-*w*Mel	Noumea, New Caledonia	2018	+	AIBF, IT, FC, IR	This study
AUS-*w*Mel	Townsville, Australia	2011*	+	BC, AIBF, IT, FC	[11]
AUS-Tet	Townsville, Australia	2016	-	IT	[10]
Bora	Bora-Bora, French Polynesia	The 90s	-	IR	[23]

BC: Backcrossing; AIBF: Artificial Infectious Blood Feeding; IT: Intrathoracic Injection; FC: Fitness Check; IR: Insecticide Resistance.

* The original line generated in 2011 has been outcrossed to Australian WT populations over time until 2016, then with AUS-Tet until now.

The Australian *w*Mel strain previously described [11,22], referred to as AUS-*w*Mel here, was used for backcrossing, and as a control for vector competence and fitness determinants analyses. The Australian tetracycline strain (AUS-Tet) is the AUS-*w*Mel strain cured of *Wolbachia* by the use of tetracycline [10]. AUS-Tet was used in comparison with the AUS-*w*Mel strain for this work for vector competence analyses.

The NC *Wolbachia* strain (NC-*w*Mel) was obtained by backcrossing AUS-*w*Mel females with NC-WT males. Six backcrosses were made in order to obtain a strain infected with *w*Mel that was genetically similar to NC-WT mosquitoes (*i*.*e*., 98% of the nuclear background similar between NC-*w*Mel and NC-WT strains). Briefly, the first backcross was made between 250 NC-WT males and 250 AUS-*w*Mel females. Then, the five following backcrosses were made between 250 NC-WT males and 250 female progeny from the previous cross. In order to ensure virginity, pupae were sexed and sorted according to size. Only virgin adults were used. *Wolbachia* frequency was checked at each generation of backcross on 160 3-5-day old females by quantitative qPCR (described below) to ensure the quality of the strain. When the six backcrosses were completed, the NC-*w*Mel strain was maintained in our laboratory.

The first generation of NC-*w*Mel strain after the completion of backcrossing (G0) was used for fitness assays. The NC-*w*Mel from generation G0 and F2 NC-WT were used for the infected blood meal experiments. NC-*w*Mel mosquitoes from generations G2-G5 and F2 NC-WT mosquitoes were used for intrathoracic injection experiments. Results were compared to those obtained for AUS-*w*Mel and a tetracycline-treated version of this line (AUS-Tet) cured of *Wolbachia* as described previously [10].

Finally, the Bora strain, an *Ae*. *aegypti* laboratory strain, was used as an insecticide-sensitive control for deltamethrin resistance tests [23].

### *Wolbachia* detection by qPCR

DNA was isolated from mosquitoes, as previously described [24]. Confirmation of *Wolbachia* infection status in mosquito tissue samples was performed using a duplex quantitative PCR targeting the *Wolbachia*-specific *wsp* gene and *Ae*. *aegypti* housekeeping *Rp*S*17* gene [25]. For each sample, qPCR was performed using a LightCycler 480 II Instrument (Roche) and the LightCycler 480 Probes Master kit (Roche).

### Assessment of virus inhibition in the NC-*w*Mel strain

In this work, artificial infectious blood meal and intrathoracic injection experiments were conducted in NC and Australia respectively.

#### Viruses and cells

All 4 DENVs, CHIKV, and ZIKV were used in this study (Table 2). Asian isolates of DENV-1 to 4 were obtained from the World Reference Center for Emerging Viruses and Arboviruses (WRCEVA). NC isolates of DENV-2 (this study), CHIKV [26], and ZIKV [27] were obtained from human sera [16] and are representative of recent arboviruses circulation in NC. Virus genotypes and origins are listed in Table 2.

**Table 2:** List of virus isolates, their origins, and infecting dose used in this study.

Strain	Genotype	Isolated	GenBank Acc. #	Obtained from	Used in	Titer used for mosquitoes infected experiments (TCID_50_ unit per mosquito)*
DENV-1	Genotype I	Vietnam 2008	FJ461335	WRCEVA	IT	1.2 x 10^5^ TCID_50_/mL (8.3 TCID_50_ units)
DENV-2	Cosmopolitan	Vietnam 2006	EU482672	WRCEVA	IT	3.4 x 10^5^ TCID_50_/mL (23.5 TCID_50_ units)
DENV-3	Genotype II	Myanmar 2008	KT452792	WRCEVA	IT	4.5 x 10^4^ TCID_50_/mL (3.1 TCID_50_ units)
DENV-4	Genotype I	Cambodia 2011	KT452802	WRCEVA	IT	8.0 x 10^5^ TCID_50_/mL (55.2 TCID_50_ units)
DENV-2	Cosmopolitan	New Caledonia 2017	MW585365	This study	AIBF	1.0 x 10^7^ FFU/mL
ZIKV	Asian	New Caledonia 2014	SRR5309452	[27]	AIBF	5.0 x 10^6^ TCID_50_/mL
CHIKV	Asian	New Caledonia 2011	HE806461	[26]	AIBF	2.0 x 10^6^ TCID_50_/mL

WRCEVA: World Reference Center for Emerging Viruses and Arboviruses; AIBF: Artificial Infectious Blood Feeding; IT: Intrathoracic injection.

* Mean TCID_50_ units per mosquito are given for IT experiments

For mosquito artificial infectious blood feeding experiments, frozen aliquots of viruses were used. Viruses’ aliquots were obtained by propagation on VERO E6 cells (kidney epithelial cells isolated from an African green monkey). Supernatants were harvested 3, 5, and 7 days after infection for CHIKV, ZIKV, and DENV respectively. For CHIKV and ZIKV, virus titers were determined by TCID_50_ on VERO E6 cells and for DENV by immune-fluorescent focus assay on *Aedes albopictus* C6/36 cells using the anti-dengue virus complex antibody, clone D3-2H2-9-21 (Millipore), and Alexa Fluor 488 goat anti-mouse IgG (Invitrogen, ThermoFisher Scientific) as previously described [16,28]. VERO E6 cells were grown at 37°C with 5% CO_2_ in Dulbecco’s Modified Eagle Medium (DMEM, Gibco, ThermoFisher) supplemented with 10% heat-inactivated fetal bovine serum (FBS, Gibco, ThermoFisher). C6/36 cells were maintained at 28°C in Leibovitz medium (Sigma-Aldrich) supplemented with 5% FBS and 10% tryptose phosphate broth (Gibco, ThermoFisher).

For mosquito intrathoracic injection experiments, viral stock production of DENVs were obtained by infection of C6/36 cells at MOI 0.1 and harvest of supernatant 7 days later. DENV concentrations were then determined by TCID_50_ on C6/36 cells using monoclonal antibody 4G2 (provided by Roy Hall), followed by incubation with HRP-conjugated secondary antibodies, and TMB substrate as described in [24]. C6/36 cells were maintained at 28°C with 5% CO2 in RPMI medium (Gibco, ThermoFisher) supplemented with 10% FBS (Gibco, ThermoFisher), 1% GlutaMAX (Gibco, ThermoFisher) and 2% HEPES (Gibco, ThermoFisher).

#### Artificial infectious blood meal

For infectious blood meals, mosquitoes were reared under the same conditions as for the maintenance of the strains (see mosquito rearing section). Five-to-7 day-old nulliparous females of NC-WT and NC-*w*Mel strains were allowed to feed for 20 minutes on a blood meal containing virus maintained at 37°C using an a Hemotek system (Hemotek Limited, Great Harwood, UK) covered with pig intestine membrane [16]. Each blood meal contained either CHIKV, ZIKV, or DENV-2 diluted to the concentrations listed in Table 2 and was supplemented with a phagostimulant (5mM ATP). Fully engorged females were then transferred into cardboard containers covered with insect netting and maintained with 10% sucrose solution at 28°C ± 1°C, 80% relative humidity under a 12 h:12 h light:dark cycle (NC laboratory standard conditions). At 3 (only for CHIKV), 7, and 14 days post-exposure, a maximum of 30 mosquitoes of each *Ae*. *aegypti* population were randomly selected. Their saliva, head, and body were collected, ground (head and body), and treated as previously described [16]. Finally, 45 μL of DMEM (for CHIKV and ZIKV) or Leibovitz medium (for DENV) were added to the collected saliva. All the samples were stored at -80°C. The detection of viral particles in each homogenate was performed by virus titration by plaque assay on VERO E6 cells for ZIKV and CHIKV and by immune-fluorescent focus assay on C6/36 cells for DENV [16,28]. The infection rate corresponds to the proportion of mosquitoes with infected bodies among all those tested. The proportion of mosquitoes with viral particles detected in saliva among all mosquitoes tested (*i*.*e*., engorged) represents the transmission efficiency.

#### Intrathoracic injection experiments

These studies were conducted in Australia and mosquitoes were reared as described in [29]. Seven-to-8 days old AUS-*w*Mel, AUS-Tet, NC-*w*Mel, and NC-WT *Ae*. *aegypti* were intrathoracically injected as previously described [24] with 69 nL of viruses diluted in RPMI to the concentrations listed in Table 2 using a microinjector (Nanoject III, Drummond Scientific) with pulled-glass capillary needles. Injected mosquitoes were then incubated for 7 days (10 mosquitoes/cup) at 26°C with 65% humidity and a 12 h:12 h light:dark cycle (Australian laboratory standard conditions) before collecting whole mosquitoes and testing them individually for infection status. To quantify viral genomic copies, total RNA was extracted from ground mosquitoes using RNeasy 96 QIAcube HT kits (QIAGEN). DENV genome copies were quantified using pan-DENV primers that bind the DENV 3’UTR [10,30] and LightCycler Multiplex RNA Virus Master (Roche) one-step qRT-PCR mix using a LightCycler 480 II Instrument (Roche).

### Fitness determinants

For fitness assays, larvae and adults were reared under the same conditions as for strain maintenance (see mosquito rearing section). Between five and eight trays for each strain were created (300 larvae for 3 L of water). After larval development, the pupae were sexed by their size and placed in cups for emergence (25 male or female pupae per cup). After adult emergence, and to guarantee that adults were virgin before crossing, solely the cups containing only males or only females were used. Different crosses have been made to evaluate MT, CI, and fertility of the different strains. Those crosses consisting of a group of 50 virgin males and 50 virgin females were performed with strains varying according to each test. Each cross was replicated three times. When adults were between 5 and 7 days old, females were blood-fed with human blood collected from donors treated with therapeutic phlebotomy (blood donor center: Service de Transfusion Sanguine, NC Hospital). The number of living females and blood-fed females were counted immediately after the blood meal. Then, 3 days after the blood meal, one egg cup with wet filter paper was placed each cage for 3 days to allow oviposition. Eggs were kept in the humid atmosphere of the insectarium for 48 h to allow embryos to fully develop before being dried. One week after egg production, filter papers were split to obtain five batches of around 200 eggs. A picture of each batch was taken and the number of eggs counted using the Mesurim Pro software (version3.4.4.0; Jean-François Madre 1995–2013).

### *Wolbachia* fitness determinants

#### Maternal transmission

To quantify the success of *Wolbachia* MT, MT crosses were set up between WT males and *Wolbachia*-infected females (crosses of NC-WT males x NC-*w*Mel females, compared to crosses of NC-WT males x AUS-*w*Mel females). One week after egg production, eggs were submerged in hatching solution separately for the three replicates of each cross. Larvae were reared to 4–6 day old adults, then 160 females of each replicate were sampled and screened by qPCR to detect *Wolbachia*.

#### Cytoplasmic incompatibility

To investigate the level of *Wolbachia*-induced CI, CI crosses were set up between *Wolbachia*-infected males and WT females (crosses of NC-*w*Mel males x NC-WT females, compared to crosses of AUS-*w*Mel males x NC-WT females). One week after the eggs were produced, five egg batches from each cage were submerged individually in hatching solution and first instar larvae counted the following day. CI was estimated by dividing the total number of hatched larvae by the total number of counted eggs for each replicate.

### Mosquito fitness determinants

#### Fertility

Fertility crosses were set up between males and females of the same strains (NC-WT males x NC-WT females; NC-*w*Mel males x NC-*w*Mel females, and AUS-*w*Mel males x AUS-*w*Mel females). The same protocol as for CI experiments was applied to the five batches of eggs from each cage. Fertility was determined by dividing the total number of 1^st^ instar larvae by the total number of eggs counted for each replicate.

#### Fecundity

Fecundity was assessed on fertility crosses. For each female strain, fecundity was estimated by the mean number of eggs laid per blood-fed female per cage.

#### Wing length

Wing length, as a proxy measurement of body size, was measured for 30 to 40 specimens of each sex and strain. The wing was removed and placed between slide and cover slip on a white surface. A picture was taken with a camera (Leica DMC2900) plugged into a stereomicroscope (Leica M205C). The measurement was made thought the LAS X software of Leica (V3.0.4). Wing length was calculated as the distance from the wing base to the wing tip.

#### Insecticide resistance

The insecticide susceptibility tests were conducted with standard WHO test tubes [31] on NC-*w*Mel, NC-WT, and Bora *Ae*. *aegypti* strains. This device allows exposing sets of 25 adult females (2–5 days old) to a filter paper impregnated with insecticide. For each dose, the insecticide was diluted in a mixture of acetone and silicone oil and 2 mL of solution was applied to each paper. Different doses of deltamethrin were tested for the resistant strains: 0% (control), 0.02%, 0.05%, 0.1%, 0.4%, and 0.9%. For the sensitive strain, doses of 0%, 0.0004%, 0.001%, 0.003%, 0.005%, and 0.01% were used. For each strain and each dose tested, four exposure tubes containing around 25 females (2–5 days old) were used. Females were exposed for 1 h. After exposure, 10% sugar solution was provided to females and mortality was recorded at 24 h.

### Statistical analysis

Statistical analysis and graphics were performed using R software (R Core Team (2017). R: A language and environment for statistical computing. R Foundation for Statistical Computing, Vienna, Austria). Comparisons of proportions were made using Fisher’s exact tests. For continuous data, the normality was assessed by group using a Shapiro-Wilk normality test. ANOVA was used to compare means across multiple groups. If any differences were found, groups were compared two by two using a Student’s t-Test. Non-parametric Kruskal-Wallis test was carried out to compare multiple groups when number of replicates was low. Comparison between two groups were made using Wilcoxon test. If multiple tests were performed, the *p*-values were adjusted using the Holm method. The statistical significance threshold for these tests was set at 0.05.

The analyses of dose-mortality responses were performed using the R script BioRssay 6.2 [32,33] as previously described [34]. Briefly, this script computes the Lethal Doses of insecticide killing 50% of the tested strains (LD_50_) and the associated confidence intervals. The comparison of strains was made by calculating the Resistance Ratios 50, or RR_50_ (= LD_50_ of tested strain/LD_50_ of the sensible strain) and their 95% confidence intervals (CI_95_). A RR_50_ in which the confidence interval does not include 1 was considered statistically significant.

## Results

### Vector competence

#### Oral challenge with infectious blood meals

**Fig 1:**
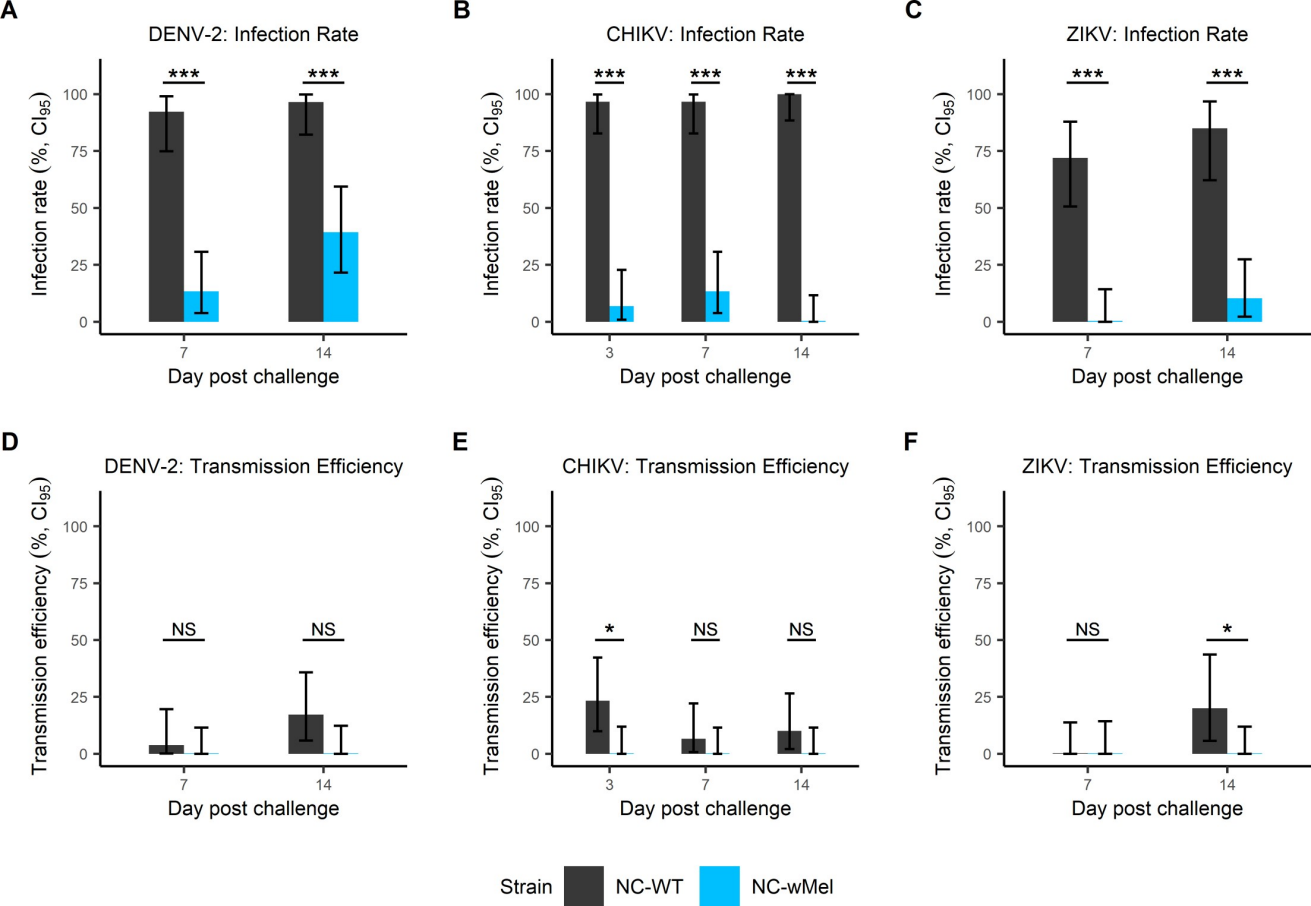
Infection rates and transmission efficiencies for NC-WT and NC-*w*Mel *Aedes aegypti* strains orally challenged with DENV-2, CHIKV or ZIKV. (A, B, C) Infection rates and (D, E, F) transmission efficiencies obtained for DENV-2, CHIKV, and ZIKV respectively at different days post-challenge. Errors bars indicate Confidence Interval at 95%. Statistically significant differences are shown in the figures (Fisher’s exact test; *: *p*-value <0.05; **: *p*-value <0.01; ***: *p*-value <0.001; NS: not significant).

NC-WT and NC-*w*Mel *Ae*. *aegypti* were orally challenged with DENV-2, CHIKV or ZIKV. Pairwise comparisons of infection rates showed that NC-*w*Mel mosquitoes were significantly less susceptible to DENV-2, CHIKV, and ZIKV compared to NC-WT *Ae*. *aegypti* irrespective of the day post infection (Fisher’s exact test, *p*-values < 0.001; Fig 1A–1C). Infection rates of NC-WT mosquitoes reached more than 90% irrespective of the day of post infection, except for ZIKV for which infection rates ranged from 72% to 85%. For NC-*w*Mel mosquitoes, infection rates ranged from 13% to 39%, 0% to 13%, and 0% to 10% for DENV-2, CHIKV, and ZIKV respectively. No infection was detected for CHIKV-exposed NC-*w*Mel *Ae*. *aegypti* at 14 days post challenge and for ZIKV-exposed NC-*w*Mel *Ae*. *aegypti* at 7 days post-challenge.

Infectious viral particles were detected in saliva of NC-WT mosquitoes for the three viruses tested irrespective of the day post infection, except for ZIKV-infected mosquitoes at 7 days post-infection (Fig 1D–1F). Overall, transmission efficiencies did not exceed 20% for NC-WT mosquitoes. No transmission was observed for NC-*w*Mel *Ae*. *aegypti* regardless of the viruses tested and the incubation time. Significant differences were observed in pairwise comparisons of transmission efficiencies between NC-WT and NC-*w*Mel mosquitoes for CHIKV at 3 days and for ZIKV and 14 days (Fisher’s exact test, *p*-values = 0.01 for CHIKV and 0.02 for ZIKV).

#### Intrathoracic injections with DENV

**Fig 2:**
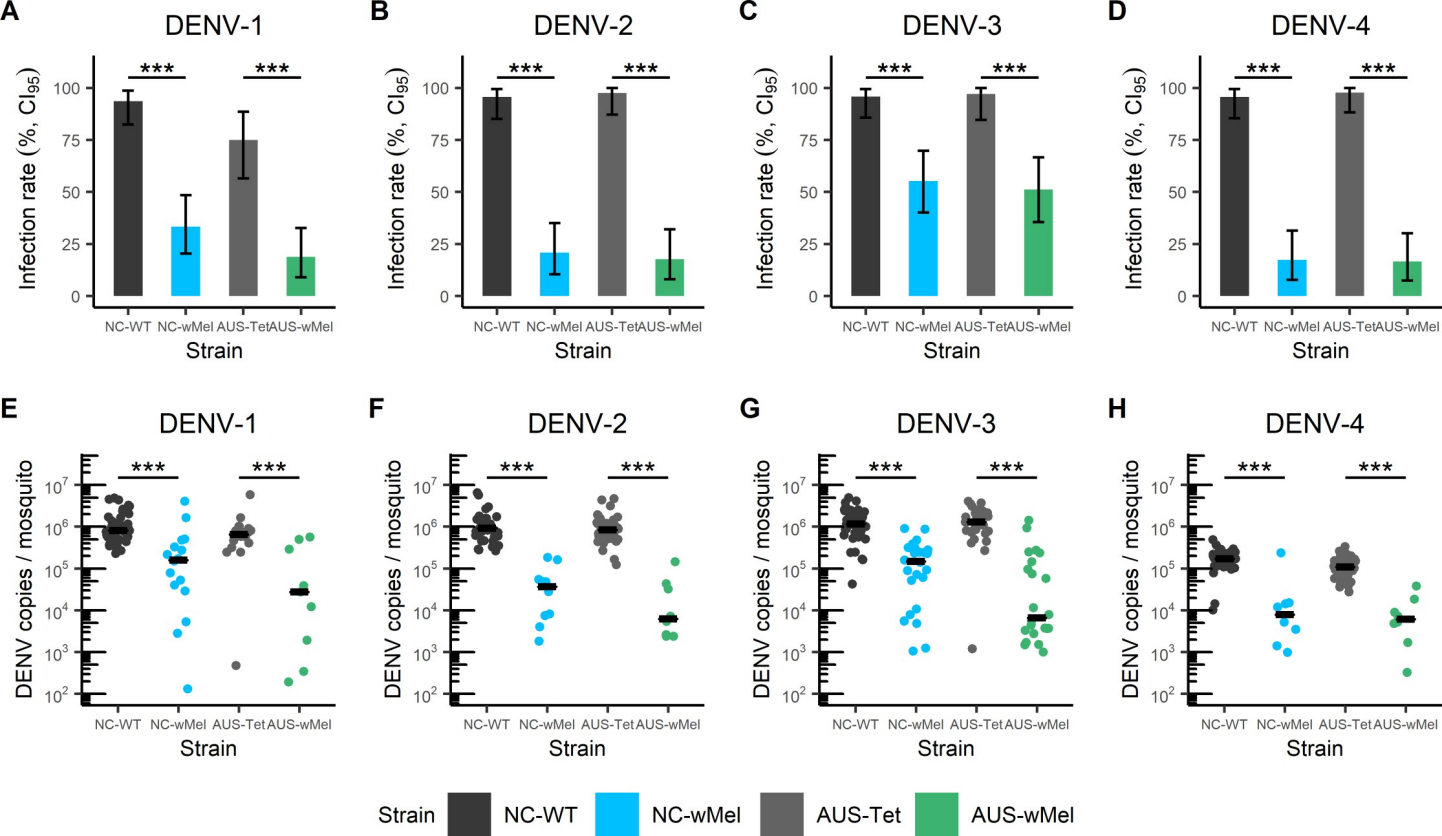
NC-WT, NC-*w*Mel, AUS-Tet and AUS-*w*Mel *Aedes aegypti* strains intrathoracically injected with the four DENV serotypes. (A, B, C, D) Infection rates obtained for DENV-1, DENV-2, DENV-3, and DENV-4 respectively at 7 days post-injection. Errors bars indicate Confidence Interval at 95%. (E, F, G, H) Viral titers obtained from infected mosquitoes at 7 days post-injection, for DENV-1, DENV-2, DENV-3, and DENV-4 respectively. Median is shown for each mosquito strain. Statistically significant differences are shown in the figures (Fisher’s exact test for infection rates; Wilcoxon test for viral titers; *: *p*-value <0.05; **: *p*-value <0.01; ***: *p*-value <0.001; NS: not significant).

NC-WT, NC-*w*Mel, AUS-Tet, and AUS-*w*Mel *Ae*. *aegypti* were injected with all four DENV serotypes. Pairwise comparisons of infection rates showed that NC-*w*Mel and AUS-*w*Mel *Ae*. *aegypti* were significantly less susceptible to DENV regardless of the serotype compared to NC-WT and AUS-Tet respectively (Fisher’s exact test, *p*-values < 0.001; Fig 2A–2D). The infection rates of NC-WT and AUS-Tet mosquitoes were above 96% for all four DENV serotypes, except for AUS-Tet mosquitoes injected with DENV-1 that was 75%. For NC-*w*Mel and AUS-*w*Mel mosquitoes injected with DENV-1, DENV-2, and DENV-4, infection rates ranged from 17% to 33%. While for DENV-3 injected mosquitoes, infection rates were higher, with 55% and 51% for NC-*w*Mel and AUS-*w*Mel *Ae*. *aegypti* respectively.

DENV viral titers in NC-*w*Mel and AUS-*w*Mel infected mosquitoes were also significantly lower compared with those obtained for NC-WT and AUS-Tet respectively regardless of the serotype (Wilcoxon test, *p*-values < 0.001; Fig 2E–2H). The medians of viral titers for NC-WT and AUS-Tet ranged from 1.1 x 10^5^ to 1.3 x 10^6^ DENV copies/mosquito, whereas the medians of DENV viral titers for NC-*w*Mel and AUS-*w*Mel ranged from 6.2 x 10^3^ to 1.6 x 10^5^ DENV copies/mosquito.

### *Wolbachia* fitness determinants

#### Maternal transmission

MT is one of the key factors for the introgression of *Wolbachia* in field mosquito populations. The MT rate measured for the NC-*w*Mel strain was high with 96% of offspring infected on average, compare with 98% for the AUS-*w*Mel strain (Table 3). No significant difference was observed between these two strains (Wilcoxon test, *p*-value = 0.7).

**Table 3:** Maternal transmission of *Wolbachia* from progeny of infected females crossed with uninfected males.

Crossing (Males x Females)	No. Replicas	Average No. parent females (range)	Average No. female progeny tested (range)	Average MT rate (range)
NC-WT x NC-*w*Mel	3	24 (22–27)	154 (142–160)	96% (96–97)
NC-WT x AUS-*w*Mel	3	24 (13–31)	160 (160–160)	98% (95–100)

#### Cytoplasmic incompatibility

CI is the second factor favouring the introgression of *Wolbachia* in field populations. For eggs obtained from the cross of NC-*w*Mel males and NC-WT females, no larvae were observed (total number of eggs = 4174). The same result was observed for AUS-*w*Mel males crossed with NC-WT females showing that NC-*w*Mel was as effective as AUS-*w*Mel males at inducing CI (total number of eggs = 2048) (Table 4).

**Table 4:** Average egg hatching rate from fertility and CI crosses.

cross type	Crossing (Males x Females)	No. Replicas	Average No. eggs tested per replica (range)	Average egg hatching rate (range)
Fertility	NC-WT x NC-WT	3	924 (851–1062)	77% (65–84)
	NC-*w*Mel x NC-*w*Mel	3	457 (256–577)	53% (47–56)
	AUS-*w*Mel x AUS-*w*Mel	3	1755 (1649–1813)	62% (56–69)
CI	NC-*w*Mel x NC-WT	3	1391 (1083–1642)	0%
	AUS-*w*Mel x NC-WT	3	683 (378–1237)	0%

### Mosquito fitness determinants

#### Fertility

In contrast to incompatible crosses, fertility crosses (males crossed with females of the same strain) produced viable eggs, with mean hatch rates of 53%, 62%, and 77% for NC-*w*Mel, AUS-*w*Mel, and NC-WT respectively (Table 4), with significant differences between crosses (Kruskal-Wallis Test, *p*-value = 0.04).

#### Fecundity

Regarding fecundity, the mean number of eggs laid per blood-fed female per cage was significantly different for all strains (Kruskal-Wallis Test, *p*-value = 0.04). The AUS-*w*Mel strain had the highest fecundity with on average 61 eggs laid per female, while NC-WT and NC-*w*Mel females laid approximately 43 and 22 eggs per females respectively (Table 5).

**Table 5:** Average number of eggs laid per female per cage.

Crossing (Males x Females)	No. Replicas	Average No. blood feed female per replica (range)	Average No. eggs per female per replica (range)
NC-WT x NC-WT	3	23 (19–29)	43 (30–56)
NC-*w*Mel x NC-*w*Mel	3	20 (17–22)	22 (15–26)
AUS-*w*Mel x AUS-*w*Mel	3	29 (28–30)	61 (55–64)

#### Wing length

**Fig 3:**
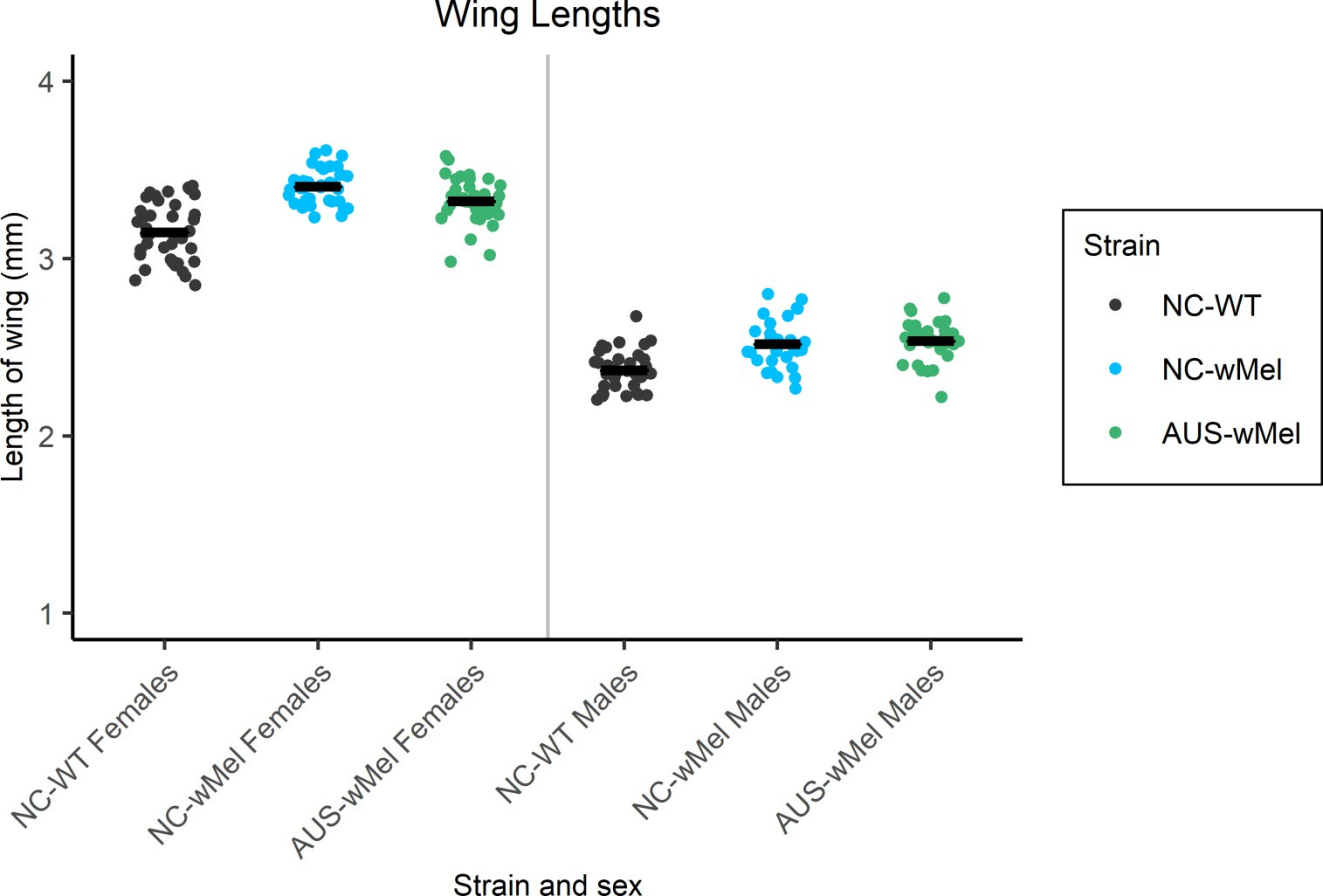
Mean wing lengths for males and females of NC-*w*Mel, AUS-*w*Mel, and NC-WT strains. Wing lengths were calculated as the distance from the wing base to the wing tip, on 30 to 40 specimens of each sex and strain. Each point represents the length of a mosquito’s wing. The black bars represent the mean of wing lengths per group.

Mean wing lengths were significantly impacted by strain and sex of the mosquitoes (ANOVA; *p-*values < 0.001). For females, all wing length means were significantly different with wing lengths of NC-WT specimens shorter (mean = 3.15 mm) when compared with *Wolbachia*-infected strains (means = 3.40 and 3.32 mm for NC-*w*Mel and AUS-*w*Mel respectively) (Student’s t-Tests; *p*-values < 0.009) (Fig 3). The same trend was observed for males, with lower average wing length for the NC-WT strain (2.37 mm) compared to both *Wolbachia*-infected strains (2.52 and 2.54 mm for NC-*w*Mel and AUS-*w*Mel respectively; Student’s t-Tests; *p*-values < 0.001). No significant difference was found between the mean wing lengths of males of the two strains carrying *Wolbachia* (Student t-Test; *p*-value = 0.53).

#### Insecticide resistance

**Fig 4:**
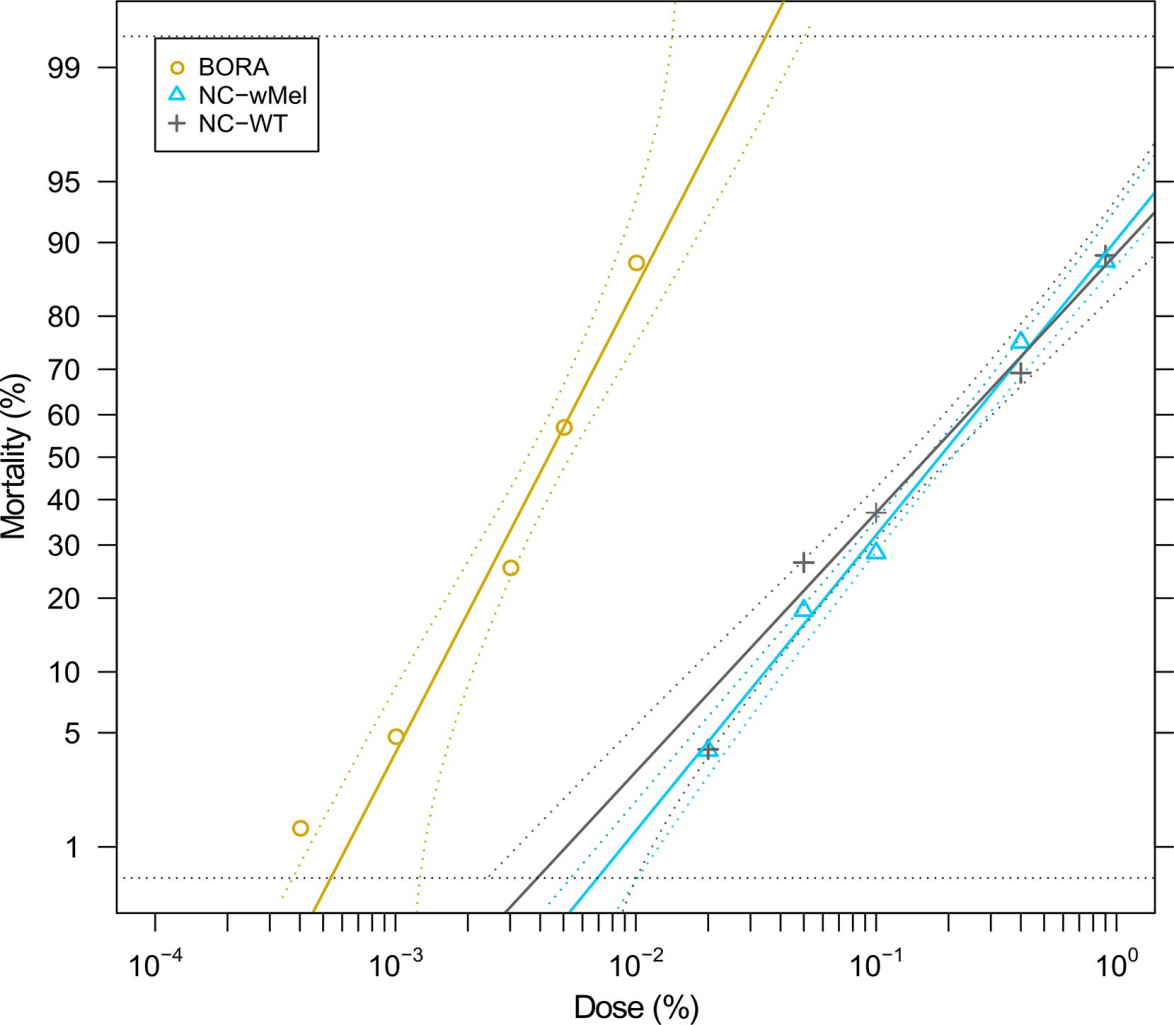
Dose-mortality to deltamethrin for NC-*w*Mel, NC-WT, and Bora strains. For each strain and each dose tested, 70 to 100 females (2–5 days old) were exposed for 1 h, and mortality was recorded at 24 h. The yellow dots (Bora), the blue triangles (NC-*w*Mel) and the gray crosses (NC-WT) represent mortalities recorded for each dose. Dotted lines indicate the Confidence Interval at 95%.

The LD_50_ was calculated for each strain, based on the mortality obtained for the six doses tested. The LD_50_ for Bora was 0.0043% of deltamethrin (CI_95_: 0.0033–0.0058%), while the LD_50_ for NC-WT and NC-*w*Mel strains raised to 0.16% (CI_95_: 0.11–0.24%), and 0.19% (CI_95_: 0.15–0.22%), respectively. The RR_50_ did not differ significantly (CI overlap) between NC-WT and NC-*w*Mel strains, with a RR_50_ of 37.9 (CI_95_: 25.4–56.6), and 42.5 (CI_95_: 26.3–68.6) for NC-WT and NC-*w*Mel respectively (Fig 4). These results together showed that there is a similar response between NC-WT and NC-*w*Mel regarding deltamethrin resistance.

## Discussion

Biological control programs involving *Wolbachia*-infected *Ae*. *aegypti* must be sufficiently robust to limit arbovirus transmission in different epidemiological settings. NC is an ideal epidemiological context for the implementation and evaluation of such a strategy as the only proven vector for dengue is *Ae*. *aegypti*, DENV outbreaks are frequent, and efficient epidemiological and entomological networks are deployed [3]. We report the development of the NC-*w*Mel *Ae*. *aegypti* strain and present results of experiments assessing its vector competence, MT, CI, fitness determinants and insecticide resistance status, as the first steps in implementing *Wolbachia* to limit arbovirus transmission in NC.

As expected, NC-*w*Mel *Ae*. *aegypti* orally challenged with DENV, ZIKV or CHIKV were significantly less susceptible to infection than WT mosquitoes. More importantly, in our study, no NC-*w*Mel *Ae*. *aegypti* were able to transmit the arboviruses tested whereas WT mosquitoes did as reported previously [16,26,28]. These data were in accordance with previous studies on pathogen blocking in *w*Mel-infected mosquitoes, which indicate that strong pathogen blocking occurs against a range of viruses [12,13,35,36]. In an intrathoracic virus challenge model, the midgut barrier is bypassed; high infection rates, and even more importantly, high dissemination rates can be achieved more quickly. NC-*w*Mel strains demonstrated significantly reduced infection rates and viral titers with all DENV tested. To our knowledge, this study is the first to assess *Wolbachia*-blocking in a recently backcrossed *w*Mel strain using both oral feeding with epidemiological relevant viruses and IT with reference viruses. By standardizing virus dose and bypassing midgut barriers to infection and dissemination, IT gives a standardized evaluation of virus blocking by *Wolbachia* infection. Conversely, oral feeding has the advantage of being more representative of the life-cycle of the virus in the vector as it recapitulates intrinsic barriers to infection. With significant results with both techniques, the findings presented here indicate the blocking observed in this new NC *w*Mel-infected *Ae*. *aegypti* strain is robust.

Even if strong virus blocking is exhibited in the NC-*w*Mel strain, its effectiveness depends on its ability to obtain high levels of introgression of *Wolbachia* in *Ae*. *aegypti* field populations via MT and CI. MT of *Wolbachia* by the new NC-*w*Mel strain of *Ae*. *aegypti* was comparable to other strains used in field deployment in other countries [10,11,37]. The *Ae*. *aegypti* NC-*w*Mel also showed complete CI. These results suggest that the NC-*w*Mel should introgress and be maintained in the field at high frequency, as shown in other trials [38].

Transinfection of *Ae*. *aegypti* by *Wolbachia* may be costly to mosquito fitness. These costs vary depending on the *Wolbachia* strain [9,10,39] and, to some extent, can negatively impact the introgression and long-term stability as observed with the *w*MelPop strain whose high fitness costs do not allow its maintenance in the field [40]. Among the various fitness parameters that we studied using the NC-*w*Mel strain, several seemed to have been impacted by *Wolbachia* infection, in particular the fecundity and fertility of females. Females of the NC-*w*Mel strain laid fewer eggs than NC-WT females and the egg hatch rate was reduced compared to NC-WT eggs. In the future, it would be interesting to increase the time for embryogenesis (*i*.*e*., before drying the eggs) for NC-*w*Mel strain in order to improve the hatching rate, as previously described [41]. The relatively low fertility and fecundity of the NC-*w*Mel strain may be due to the cumulative fitness costs of *Wolbachia* and insecticide resistance. In fact, this phenomenon has already been observed in Brazil where a strain highly resistant to insecticides exhibited fecundity and hatch rates close to those we observed in our study [42]. These levels of *Wolbachia* cost on the fertility and fecundity of *Ae*. *aegypti* have already been observed in Australian and Brazilian strains, without impacting the establishment of *w*Mel in field populations [38,42]. The number of replicates on the fecundity and fertility experiments was limited. They should be repeated on isolated female mosquitoes to ensure that this cost does not limit the installation of *Wolbachia* in the field.

Other fitness parameters were evaluated. No reduction in wing size was observed in mosquitoes infected with *w*Mel. In contrast, a slight, but significant, increase in the size of the wings of males and females of *w*Mel-infected strains (NC and AUS) was observed. Although this difference in wing size between the NC-WT and AUS-*w*Mel strains could be due to a genetic background difference, it is less likely that wing-size difference between the NC-WT and NC-*w*Mel strains relies on genetic background differences. Indeed, the NC-*w*Mel strain was obtained through six generations of backcrossing with the NC-WT strain; these two strains therefore share a large part of their nuclear genome. This observed increase in wing size is consistent with previous work by [39] in which a similar increase in the size of *Wolbachia*-infected adults was observed, which was potentially due to a longer duration of larval development and a positive impact of *Wolbachia*.

The last fitness component that can negatively impact the establishment of *Wolbachia* in the field is a difference in insecticide resistance levels. If insecticide treatments are applied, a susceptible mosquito strain will be disadvantaged compared with resistant WT mosquitoes. This phenomenon was observed in Brazil, where the first *Wolbachia*-infected *Ae*. *aegypti* strain released exhibited much lower resistance levels than the field populations. As a result, *Wolbachia* did not establish in this first trial, necessitating the generation of a new insecticide resistant strain [42]. To avoid this problem, the NC-WT strain was generated through backcrossing with a field strain resistant to deltamethrin, the only insecticide currently used in Noumea. The level of resistance to deltamethrin was similar between NC-*w*Mel strain and WT field mosquitoes, which should allow *Wolbachia* establishment in Noumea, even in the presence of insecticide treatments.

Such an operational program needs to be carefully monitored to assess the evolution of *w*Mel *Ae*. *aegypti* strain in the environment. Abiotic and biotic factors could impact the strategy, particularly viral adaptative evolution and specific environmental conditions that may impact *Wolbachia* efficiency in the long term [43]. In the long-term, DENV genetic adaptation is eventually expected. This could reduce complete blocking by *Wolbachia* but partial DENV blocking should persist indefinitely [44], still reducing the risk of dengue outbreaks. Concerning environmental conditions, heat stress caused by elevated ambient temperatures (above 30°C during the day in summer in Noumea) could have a negative impact on *Wolbachia* density [45]. However, it should be transient [46] given the temperatures recorded in Noumea (25°C in average in dry season) [47]. The strategy could also be affected by the introduction of new species of mosquitoes, especially those transmitting arboviruses as different species have been recorded in neighboring islands [48,49]. In New Caledonia, *Aedes scutellaris* has been detected between March 2016 and December 2017 [50,51]. Since then, no new detection occurred despite a regular monitoring specifically dedicated to this species. To reduce the risk of introduction, a regular monitoring of the main international entry points is managed by the Department of Health and Social Affairs of New Caledonia in the framework of the International Sanitary Regulations.

Despite the different factors which could influence the strategy, results now available from the field show that the method is stable after several months [25] to several years [52]. Furthermore, the Vector Control Advisory Group (WHO) has recently concluded that “*w*Mel introgression into populations of *Ae*. *aegypti* demonstrates public health value against dengue” [53]. As *Ae*. *aegypti* is the only proven vector for dengue in NC, we expect a high efficiency of the method on the reduction of dengue and other arboviruses cases in Noumea as recently shown in Yogyakarta, Indonesia [18].
